# Endogenously regulated Dab2 worsens inflammatory injury in experimental autoimmune encephalomyelitis

**DOI:** 10.1186/2051-5960-1-32

**Published:** 2013-07-09

**Authors:** Vilija G Jokubaitis, Melissa M Gresle, Dennis A Kemper, William Doherty, Victoria M Perreau, Tania L Cipriani, Anna Jonas, Gerry Shaw, Tanja Kuhlmann, Trevor J Kilpatrick, Helmut Butzkueven

**Affiliations:** 1 The Florey Institute of Neuroscience and Mental Health, University of Melbourne, Parkville, Victoria, Australia; 2 Department of Anatomy and Neuroscience, University of Melbourne, Parkville, Victoria, Australia; 3 Department of Medicine (RMH), University of Melbourne, Parkville, Victoria, Australia; 4 Department of Neuroscience, McKnight Brain Institute, University of Florida College of Medicine, Gainesville, Florida, USA; 5 EnCor Biotechnology Inc. Gainesville, Gainesville, Florida, USA; 6 Institute of Neuropathology, University Hospital Münster, Pottkamp 2, 48149, Münster, Germany

**Keywords:** Disabled-2, Dab2, Experimental autoimmune encephalomyelitis, Multiple sclerosis, Neuroinflammation

## Abstract

**Background:**

Neuroinflammation regulates both disease pathogenesis and repair in multiple sclerosis. In early multiple sclerosis lesion development, neuroinflammation causes demyelination and axonal injury, the likely final common determinant of disability. Here we report the identification of a novel neuroinflammatory mediator, Disabled-2 (Dab2). Dab2 is an intracellular adaptor protein with previously unknown function in the central nervous system.

**Results:**

We report that Dab2 is up-regulated in lesional macrophages/microglia in the spinal cord in murine experimental autoimmune encephalomyelitis, a model of multiple sclerosis. We demonstrate that *dab2* expression is positively correlated with experimental autoimmune encephalomyelitis disease severity during the acute disease phase. Furthermore, *dab2*-deficient mice have a less severe experimental autoimmune encephalomyelitis disease course and suffer less neuroinflammation and less axonal injury than their wild-type littermates. We demonstrate that *dab2* expression is strongly associated with the expression of inducible nitric oxide synthase. We further demonstrate that Dab2 is expressed at the protein level by macrophages in early acute human multiple sclerosis lesions and that this correlates with axonal injury.

**Conclusions:**

Together, these results suggest that endogenous Dab2 exacerbates central nervous system inflammation, potentially acting to up-regulate reactive oxygen species expression in macrophages and microglia, and that it is of potential pathogenic relevance in Multiple Sclerosis.

## Background

Neuroinflammation regulates both disease pathogenesis and repair in multiple sclerosis (MS). In acute MS lesions, neuroinflammation causes demyelination [1] and axonal injury [2,3]. A better molecular understanding of the MS-associated neuroinflammatory process is likely to yield novel proteins and pathways that could enhance our understanding of pathogenesis or yield novel therapeutic targets. Using a common animal model of MS, experimental autoimmune encephalomyelitis (EAE), our laboratory sought to identify genes that are differentially expressed by glia in EAE relative to healthy mice, and that could potentially regulate neuroinflammatory processes. Using RNA microarray expression analysis we identified Disabled-2 (Dab2) as being significantly up-regulated in EAE and, based on extant literature, potentially expressed by glia, and therefore chose this molecule for further study.

Disabled-2, a cytosolic adaptor protein, is part of a larger family of proteins comprising *Drosophila* Disabled (Dab) and mammalian Disabled-1 (Dab1). Unlike Dab and Dab1, which have neuronally-restricted expression profiles [4,5], Dab2 is broadly expressed in the brain, kidney, ovaries, breast and other organs [6-9]. There are two predominant Dab2 splice variants encoding 96 kD (p96) and 67kD (p67) proteins [9]. The p96 Dab2 isoform is the predominant isoform found in the adult, whereas, the p67 isoform is predominantly expressed during embryogenesis [10]. Disabled-2 expression within the visceral endoderm during embryonic development is necessary for survival of the early embryo [8,10,11]. The Dab-2 has also been shown to be involved in numerous other biological functions, varying according to cell type, cell cycle stage and developmental stage, including receptor endocytosis [10,12,13] and cell migration [8,14].

Additionally, Dab2 has previously been shown to be up-regulated during various CNS injury responses. For instance, a study of mechanical cryoinjury within the rat frontal cortex reported the up-regulation of the p96 Dab2 isoform by macrophages and astrocytes within lesioned tissue [15]. Furthermore, it has previously been reported that *dab2* is up-regulated within human MS lesions, in a microarray analysis of autopsy specimens [6]. However, its role or function in this context remains to be elucidated.

We show that *dab2* expression is positively correlated with EAE disease severity during the acute disease phase, and that Dab2 is principally expressed by microglia/macrophages and astrocytes within EAE lesions. Using Dab2 knock-out mice, we demonstrate that, in the absence of Dab2, EAE disease severity is ameliorated and that this correlates with a decrease in the expression of inducible nitric oxide synthase (*iNOS)* and interleukin-1β (*IL-1β*). In Multiple Sclerosis, we show that Dab2 is highly expressed in macrophages in the early acute lesion, but that its expression is diminished in late acute lesions and almost absent in chronic MS lesions. Together these data demonstrate that endogenous Dab2 expression exacerbates EAE severity, and is of potential relevance to MS pathology because its macrophage expression profile is associated with lesion acuity.

## Results

### Dab2 Expression is up-regulated in murine EAE spinal cord

We initially sought to identify genes that could potentially regulate neural cell survival or repair processes in the context of inflammatory demyelination. Therefore, a comparative analysis of gene expression was undertaken in healthy control SJL/J mice and those subjected to moderate EAE (clinical grade 2.5). Microarray analyses revealed that *dab2* mRNA expression was consistently up-regulated in the disease state on average 3.5 fold (p=2.05x10^-3^; supplementary data).

**Figure 1:**
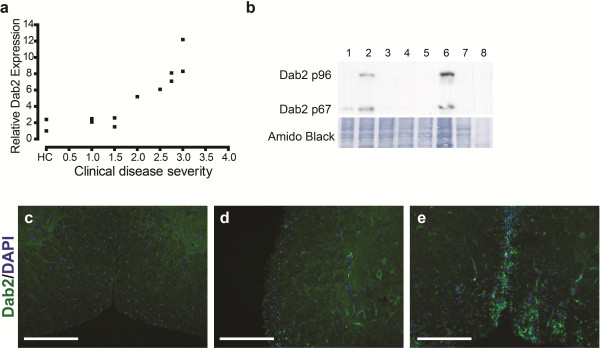
**Dab2 expression is up-regulated in the murine C57B/6 MOG-EAE model relative to health. a**: Realtime PCR quantitation of dab2 gene expression in the spinal cords of MOG EAE-immunised mice was expressed relative to healthy controls. Analysis reveals that dab2 gene expression is positively correlated with disease severity (HC: Healthy Control; Spearmann’s rank correlation r2=0.921, p<0.0001). **b**: Western blot analysis of Dab2 expression in healthy control mice and those subjected to EAE (grade 3.0). 1. Healthy spinal cord 2. EAE spinal cord 3. Healthy forebrain 4. EAE forebrain 5. Healthy hindbrain 6. EAE hindbrain 7. Healthy spleen 8. EAE spleen. Shown is a marked up-regulation of both the p96 and p67 Dab2 isoforms in the EAE spinal cord and hindbrain. **c**/**d/e**: Scale bar represents 200μm **c**: Dab2 staining is undetectable in the healthy spinal cord. **d**: A region of EAE spinal cord spared from inflammation shows no Dab2 staining. **e**: A region of EAE spinal cord containing inflammatory infiltrates (DAPI-positive nuclear accumulation) shows Dab2 staining throughout the lesions.

To independently confirm these microarray findings, we assessed Dab2 gene and protein expression in C57B/6 mice during MOG-induced EAE. Quantitative PCR analysis revealed a strong, positive correlation between *dab2* expression and EAE disease severity with a Spearman’s rank co-efficient of 0.92, p<0.0001 (Figure 1a). In order to determine whether this up-regulation of *dab2* at the mRNA level translated into an increase in Dab2 protein, Western Blot analyses of relevant tissues were performed. Analysis of tissue derived from healthy control mice and mice with an EAE grade of 3.0 showed that Dab2 was markedly up-regulated in the spinal cord and hindbrain, but not forebrain of EAE mice, consistent with the pattern of immune-mediated injury in this EAE model. Notably, Dab2 expression was not detectable in the spleen of healthy control or EAE animals (Figure 1b) indicating that Dab2 is not highly expressed in lymphocytes or resting macrophages. Immunohistochemistry was then performed on coronal sections of spinal cord to characterize the pattern of Dab2 expression in health and disease. Concordant with the quantitative PCR and Western Blot data, Dab2 expression could not be detected in the spinal cords of healthy mice (Figure 1c), however, Dab2 staining increased with EAE disease severity, where it was most intense from clinical grade 2.5. No Dab2 staining was detectable in the normal appearing white matter (NAWM) of the EAE spinal cord (Figure 1d). We found that Dab2 expression was predominantly interspersed throughout inflammatory lesions (Figure 1e). Staining was most intense in the core of inflammatory lesions, and tapered off towards their edges.

**Figure 2:**
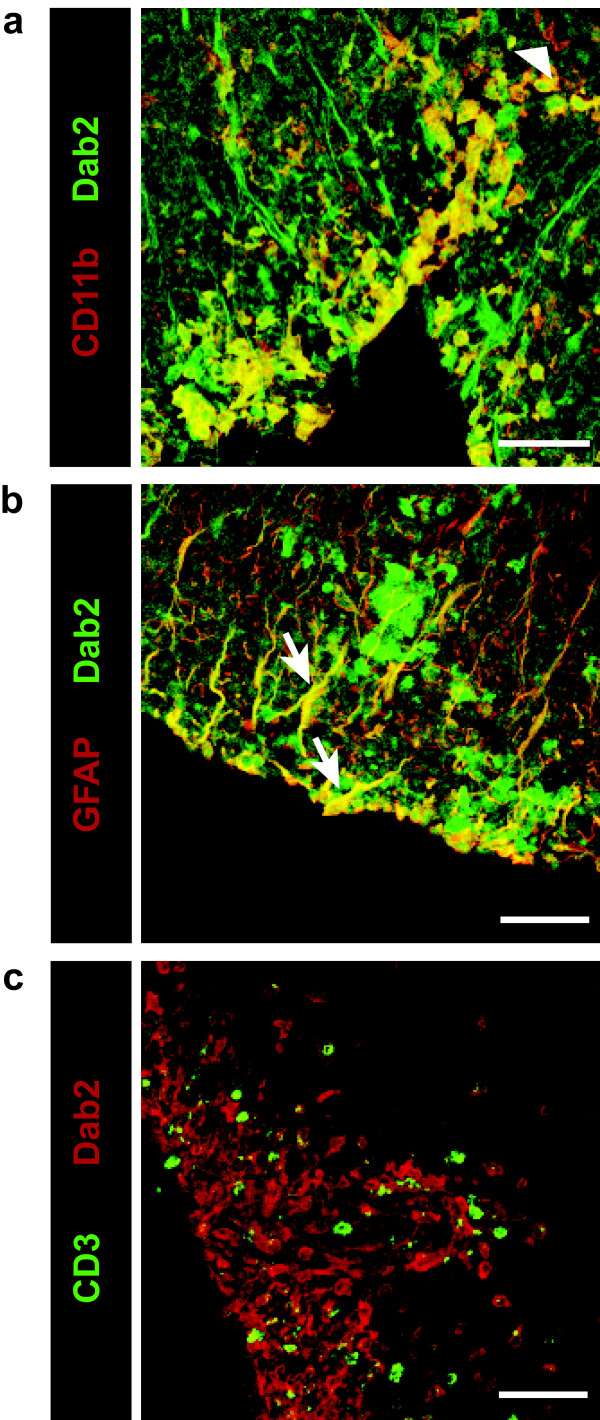
**Characterization of Dab2 expression within the EAE spinal cord.** Immunoflourescent staining for Dab2 in the lumbar expansion in an EAE grade 3.0 C57B/6 wild-type mouse spinal cord. Representative confocal images show **a**: Dual epifluorescence image showing that the vast majority of CD11b-positive macrophages/microglia also stain positively for Dab2. Dab2-negative microglia (arrowhead). **b**: Dual epifluoresence image showing that a large proportion of GFAP-positive astrocytes also stain positively for Dab2 (arrows). **c**: Dual epifluoresence image showing that CD3-positive T-lymphocytes do not express Dab2 within inflammatory infiltrates. Scale bars represent 50 μm.

To determine the cell specificity of Dab2 expression within EAE lesions, dual epifluorescence immunohistochemical analyses were performed using confocal microscopy. The Dab2 protein was predominantly expressed by CD11b-positive macrophages/microglia (Figure 2a) and GFAP-positive astrocytes (Figure 2b) as well as a small sub-population of PLP-positive oligodendrocytes and NG2-positive oligodendrocyte progenitors (Additional file 1: Figure S1). However, Dab2 was not expressed by CD3-positive T-cells (Figure 2c); demonstrating that the up-regulation of Dab2 expression in the C57B/6 EAE spinal cord is a macrophage/microglial and macroglial cell response. Using primary cells derived from early post-natal rats, we confirmed that *dab2* and Dab2 are more highly expressed by microglia and astrocytes than either oligodendrocytes or their progenitors in their native state *in vitro* (Additional file 2: Figure S2).

### Deletion of Dab2 expression reduces EAE severity

**Figure 3:**
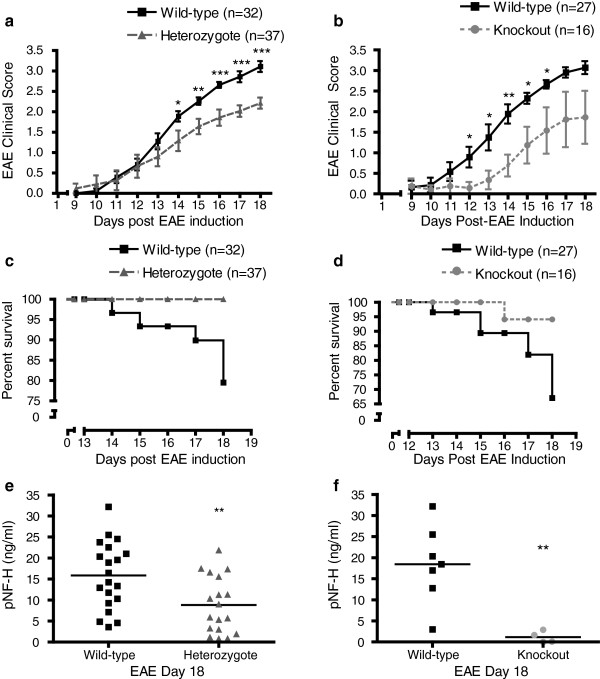
**Dab2 deletion ameliorates EAE disease severity. a/b**: EAE clinical scores of MOG-induced C57B/6 wild-type, Dab2 heterozygote and Dab2 knockout mice. **a**: Mice heterozygous for Dab2 have significantly repressed EAE disease scores from 14 days post-EAE induction (Mann–Whitney rank sum test; *p<0.05, **p<0.01, ***p<0.001) **b**: Dab2 knockout mice have significantly repressed EAE disease scores from 12 days post-EAE induction (Mann–Whitney rank sum test; *p<0.05, **p<0.01) c/d: Kaplan-Meier survival curves for wild-type, Dab2 heterozygote and Dab2 knockout mice. **c:** Figure shows that one hundred percent Dab2 heterozygote mice survived the EAE insult relative to eighty-one percent of wild-type mice (Chi-Square test, p<0.01). **d**: Figure shows that ninety-six percent of Dab2 knockout mice survived the EAE insult realtive to sixty-seven percent of wild-type mice (Chi-Square test, p<0.05). **e/f**: Serum phosphorylated neurofilament heavy chain (pNF-H) ELISA measure of axonal injury. **e**: Figure shows that mice heterozygous for Dab2 have a significantly lower concentration of pNF-H in their serum than wild-type mice (Two-tailed Student’s t-test p<0.01). **f**: Figure shows that Dab2 knockout mice have significantly less pNF-H in their serum than wild-type mice (Two-tailed Student’s t-test, p<0.01).

To ascertain the impact of Dab2 expression on the disease course of MOG-EAE, we induced the disease in Dab2 wild-type, Dab2 heterozygote and Dab2 knockout littermates [8]. The Dab2 wild-type and heterozygous mice had a similar disease onset, however, EAE scores of Dab2 heterozygous mice diverged from those of wild-type littermate controls from 14 days post-immunization and remained significantly reduced until experimental end-point (Figure 3a). Mice with Dab2 deletion had disease scores that significantly diverged from 12 days until 16 days post-immunization (Figure 3b). Both Dab2 heterozygote and Dab2 knockout mice displayed significantly greater survival rates in response to EAE challenge relative to Dab2 wild-type littermate controls (Chi-Square test p<0.01, p<0.05 respectively; Figure 3c &3d).

It has now been established that MOG-EAE features high levels of lesional inflammatory axonal injury [16-18]. To determine the effect of Dab2 deletion on axonal injury, serum was collected from mice at disease end-point and analysed for phosphorylated neurofilament heavy chain (pNF-H) concentration by ELISA. Analyses revealed that both Dab2 heterozygote (Figure 3e) and Dab2 knockout (Figure 3f) mice had significantly less pNF-H in their serum (p<0.01 for both), and hence suffered less axonal injury than their Dab2 wild-type littermates.

**Figure 4:**
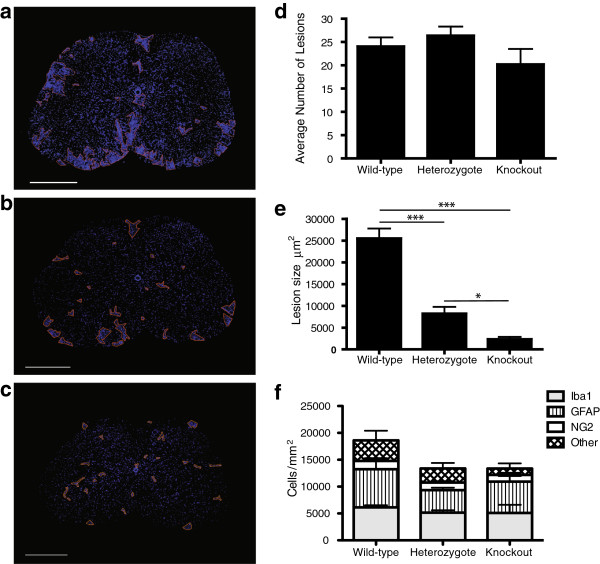
**Histological analysis of the lumbar expansion in Dab2wt/wt, Dab2wt/ko and Dab2ko/ko mice subjected to EAE.** Representative sections of the lumbar expansion of the spinal cord of **a**: a wild-type mouse subjected to EAE **b**: a Dab2 heterozygote mouse subjected to EAE **c**: a Dab2 knockout mouse subjected to EAE. **a/b/c**: Orange lines outline immune infiltrates. Scale bar represents 500μm **d**: Analysis of average number of immune lesions in the spinal cords of Dab2 heterozygous and knockout mice relative to wild-type controls reveals that mice heterozygous for Dab2 and knockout for Dab2 have a similar number of lesions to wild-type mice (p>0.05) **e**: Analysis of average infiltrate area reveals that the size of immune infiltrates found in the lumbar expansion of Dab2 heterozygous mice and Dab2 knockout mice is significantly smaller than those found in the lumbar expansion of wild-type mice (***p<0.001) Similarly, the average size of immune infiltrates in the spinal cords of Dab2 knockout mice is significantly smaller those found in Dab2 heterozygous mice (*p<0.05). **f**: Cell densitometry analysis within the core of EAE lesions reveals that there are no significant differences between the proportional composition of various glial cell types within lesions in wild-type and Dab2 heterozygous mice, however, total cell numbers per lesion are significantly less in Dab2 heterozygous and knockout lesions (**p<0.01). Average ± SEM; One-Way ANOVA, Tukey’s post-hoc test; n ≥ 4 mice/genotype for all above analyses.

It has been demonstrated that Dab2 can regulate the migration of various cell types both *in vivo* and *in vitro*[8,14] thus raising the possibility that the EAE phenotype of Dab2 heterozygous and knockout mice could be due to a recruitment block of macrophages into the EAE spinal cord, and subsequently of macrophages/microglia into inflammatory lesions. To examine this possibility, we assessed the inflammatory infiltrates contained within the spinal cords of Dab2 wild-type, heterozygous and knockout EAE mice (Figure 4a-c). There were no significant differences in the average number of inflammatory infiltrates within coronal sections of the lumbar expansion of Dab2 wild-type, heterozygote or knockout mice (24±2 lesions/section, 26±2 lesions/section, 20±3 lesions/section respectively; Figure 4d). However, the average size of lesions differed significantly between genotypes (25,400 μm^2^ ± 2,300 μm^2^ wild-type; 8,200 μm^2^ ± 1,500 μm^2^ heterozygote; 2310μm^2^ ± 510 μm^2^ knockout; Figure 4e). Densitometry analyses of cell types shown to express Dab2 (macrophages/microglia, astrocytes and NG2 positive cells) were then performed to further characterise the composition of EAE lesions. It was found that Dab2 wild-type, Dab2 heterozygote and Dab2 knockout lesions contained proportionally equivalent numbers of macrophages/microglia, astrocytes, NG2 progenitors and other cell types, however, Dab2 heterozygote and knockout lesions were less cell dense overall than lesions found in the spinal cords of Dab2 wild-type mice (Figure 4f). Similarly, normal-appearing white matter (NAWM) in Dab2-deficient mice was less cell-dense than NAWM from wild-type mice, with no differences in cell-type proportions (Additional file 3: Figure S3A). In contrast, cell density and cell-type proportions were equivalent in healthy tissue derived from Dab2 wild-type, and Dab2-deficient mice (Additional file 3: Figure S3B) arguing that the observed EAE phenotype was not driven by differences in neural tissue composition at baseline.

### EAE can be induced in C57B/6 mice by Dab2-deficient T-cells

To exclude the possibility that EAE disease severity was ameliorated in Dab2 deficient mice due to aberrant T-cell priming or activation, passive transfer EAE experiments were performed (Additional file 4: Figure S4). We found that disease could be transferred from both Dab2 heterozygous and Dab2 knockout mice to Dab2 wild-type recipient (C57B/6) mice. Disease was induced in 5/5 wild-type recipients after transfer of T-cells from Dab2 knockout mice (EAE day 22 average grade 1.25; range 1–2.25) and in 3/3 wild-type recipients after transfer of T-cells from Dab2 heterozygote mice (EAE day 22 average grade 1.17; range 1–1.5). These results collectively suggest that the genetic deletion of Dab2 does not significantly interfere with peripheral T-cell priming, activation or initial lesion formation.

### Dab2 Gene expression is positively correlated with iNOS and IL-1β gene expression in mice subjected to EAE

**Figure 5:**
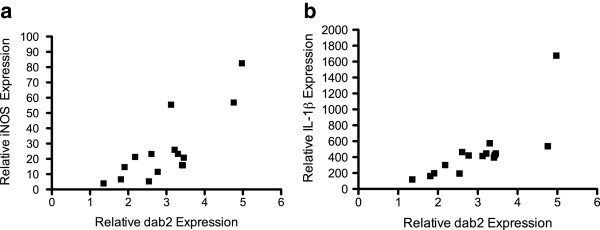
**qPCR quantification of gene expression in the lumbar expansion of spinal cords of Dab2wt/wt mice (n=15) in acute EAE.** Multivariate linear regression analysis reveals that Dab2 gene expression is positively correlated with **a**: iNOS gene expression (adjusted r^2^=0.65, p=0.003) and **b**: IL-1β gene expression (adjusted r^2^=0.45, p=0.015) when controlling for disease grade 18 days post-EAE induction.

Given that Dab2-deficient mice did not display a macrophage recruitment deficit to lesions, we explored the possibility that Dab2 could regulate EAE disease pathogenesis by altering microglial/macrophage activation. In EAE, activated microglia/macrophages secrete a wide variety of factors including inflammatory cytokines, reactive oxygen species and trophic factors which contribute to disease pathogenesis [19,20]. We performed a targeted quantitative PCR analysis of IFNγ, IL-1β, TGFβ, TNFα and iNOS gene expression in Dab2 wild-type and Dab2-deficient mice in EAE spinal cord tissue harvested at day 18. Using multivariable linear regression analysis, we found that *dab2* gene expression was positively correlated with *iNOS* (adjusted r^2^=0.65, p=0.003; Figure 5a) and *IL-1β* (adjusted r^2^ = 0.45, p=0.015; Figure 5b) gene expression independent of disease grade, whereas other tested gene expression profiles were not correlated with Dab2 levels when adjusted for grade. These results strongly suggest that Dab2 could worsen EAE lesion evolution by altering microglial/macrophage activation through the regulation of iNOS and IL-1β signalling pathways.

### Biochemical responses to inflammatory stimuli are altered in Dab2-deficient macrophages

To examine whether Dab2-deficient macrophages have altered responses to pro-inflammatory stimuli, we treated bone marrow macrophages (BMM) derived from Dab2 heterozygote and Dab2 wild-type mice with 1 μg/mL lipopolysaccharide (LPS) or with PBS. Three independent qPCR experiments showed that Dab2 heterozygote BMM expressed between 35 and 300 fold less iNOS six hours post LPS stimulation than wild-type control BMM. However, we did not see a corresponding decrease in IL-1β production under these same experimental conditions. These results support the hypothesis that *dab2* expression by cells of the macrophage/microglial lineage is associated with the production of nitric oxide and provides a putative mechanism through which Dab2 expression could regulate axonal injury in EAE.

### Dab2 Responses to stimulation by exogenous cytokines *in vitro*

Using a candidate approach, we aimed to identify factors that could induce *dab2* and Dab2 expression. We tested three candidate molecules reported to regulate Dab2 expression in various cell types *in vitro* including TGFβ_1_[21,22], *all-trans* retinoic acid [23,24] and IFNγ [14]. We additionally looked at *dab2* expression in response to the pro-inflammatory mediator lipopolysaccharide (LPS). To identify putative modulators of *dab2* and Dab2, we performed *in vitro* analyses in primary microglia derived from early post-natal rats. We found that *dab2* expression was in fact diminished in response to all-*trans* retinoic acid and TGFβ_1_ treatment. Triplicate experiments showed that *dab2* gene expression was down-regulated in response to 1μM all-*trans* retinoic acid at 6 hours (0.46 ± 0.09, p = 0.002) and 24 hours (0.45 ± 0.03, p = 0.004) post-treatment relative to control expression (1.02 ± 0.08). In addition we found that *TNFα* expression was similarly diminished in response to all-*trans* retinoic acid (Additional file 5: Figure S5a). Similarly, *dab2* gene expression levels were reduced at 6 hours (0.46 ± 0.08, p = 0.014) and at 24 hours (0.37 ± 0.03, p = 0.004) relative to control expression (1.03 ± 0.08) in response to 10 ng/ml TGFβ_1_ (Additional file 5: Figure S5b). Again, we found this to be a likely non-specific anti-inflammatory response with *TNFα* expression levels also reduced in the experiment. In addition, we found that Dab2 was phosphorylated in response to TGFβ_1_ treatment. Phosphorylation of Ser24 was rapid, but transient, lasting less than 30 minutes (Additional file 5: Figure S5c). We were unable to find any cytokines that induced *dab2* gene expression, including IFNγ and LPS (data not shown).

### Dab2 Is highly expressed in acutely demyelinating human MS lesions

**Figure 6:**
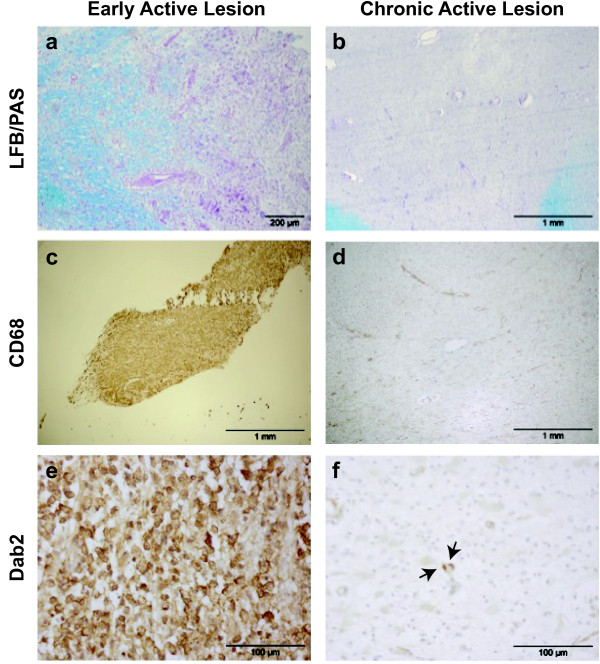
**Characterization of Dab2 expression within MS lesions.** Immunohistochemical staining of an early acitve lesion (biopsy) and a chronic active lesion. **a/b**: Luxol Fast Blue/Periodic Acid Schiff stain showing regions of demyelination (pink). **c/d**: Staining for the macrophage/microglial marker CD68 (brown) showing large numbers of macrophages throughtout both lesion types. **e/f**: Dab2 staining in the early acitve and chronic active lesion. **e**: Dab2 staining can be seen throughout the early active lesion expressed by cells of a macrophage morphology. **f**: Very little perivenular Dab2 staining (arrows) can be found in the chronic active lesion.

Having demonstrated that, in EAE spinal cord Dab2 is expressed predominantly by macrophages/microglia and astrocytes, we sought to characterise Dab2 expression within MS lesions (Figure 6). Disabled-2 expression was assessed in early active (acute), late active (acute), or chronic active lesions, classified according to previously described criteria [25]. Acute active lesions were defined by the presence of macrophages with MOG-positive myelin debris (early active) or PAS-positive but MOG-negative myelin debris (late active), (Figure 6c &6d). Chronic active lesions had a rim of macrophages/microglia surrounding a hypocellular demyelinated lesional core.

Immunohistochemical analysis revealed that Dab2 was very highly expressed within early active lesions (Figure 6e) and coincided with intense APP staining (Additional file 6: Figure S6), a marker of axonal injury (Table 1). Examination of a late acute lesion revealed a marked decrease in Dab2 staining, as well as a decrease in the number of APP-positive elements. Occasional Dab2-positive cells were found within chronic active lesions (Figure 6f), more commonly in the venous spaces and only rarely in the parenchyma (Table 1). Morphologically, the dab2-expressing cells were characterised as macrophages or microglia by an experienced neuropathologist (TK).

**Table 1:** Immunoreactivity in human MS Lesions

	**Marker (μ ± SD)**		
**Lesion type**	***Dab2 +ve cells2***	***CD68 +ve cells***	***APP +ve elements***
Early active – biopsy 1	129.9 ± 25.5	164.1 ±28.3	24.2 ± 5.8
Early active – biopsy 2	86.4 ± 7.7	96.9 ± 12.3	23 ± 4.2
Late active – biopsy 3	10.1 ± 4.1	96.9 ± 19.7	4.9 ± 3.6
Chronic active – autopsy 1	0.64 ± 1.0	58.7 ± 15.8	4.2 ± 1.9
Chronic active – autopsy 2	0.20 ± 0.42	14.0 ± 4.1	2.6 ± 0.97
Chronic active – autopsy 3	0.10 ± 0.32	12.8 ± 4.1	1.8 ± 1.2

Table 1 summarizes Dab2, CD68 and APP positive cell density in serial sections of lesions obtained from biopsy or autopsy specimens.

## Discussion

In the present study, we have demonstrated that *dab2* expression in the spinal cord is induced by EAE and positively correlated with EAE disease severity during the acute disease phase. Expression of the protein in this context is restricted to macrophages/microglia, astrocytes and oligodendrocytes, particularly perilesional and lesional microglia/macrophages and astrocytes. A recent study has similarly demonstrated that Dab2 is expressed by astrocytes and macrophages in the rat EAE model [26].

We have shown that mice with a heterozygous or homozygous deletion of *dab2* exhibited lower disease severity and higher survival rates, less axonal injury and smaller neuroinflammatory lesions when subjected to EAE compared to wild-type littermate controls. The level of induced *dab2* gene expression was positively correlated with the gene expression of *iNOS* and *IL-1β*, markers of microglial/macrophage activation [19], after adjusting for disease severity. A direct link between *dab2* induction and expression of *iNOS* was further supported by our *in vitro* analyses that showed that *dab2*-deficient macrophages expressed lower levels of *iNOS* relative to wild-type controls when challenged with LPS, activating TOLL-like receptors. Therefore, these results suggest that Dab2 upregulation is associated with a pro-inflammatory M1 macrophage/microglial phenotype.

It is most likely, therefore, that Dab2 regulates microglial/macrophage activation, and thereby lesion expansion, via increased expression of nitric oxide (NO) and, potentially, IL-1β in the context of murine EAE. These findings are particularly interesting as nitric oxide has been directly implicated in oligodendrocyte cell death [27,28] and has previously been reported to play a role in MS lesion pathogenesis [29-32]. Specifically, nitric oxide and iNOS producing microglia/macrophages and astrocytes are present in active human MS lesions, but are rare in chronic lesions and absent in normal tissue, supporting the notion that macrophage and astrocyte-derived nitric oxide production also exacerbate MS lesion-associated tissue injury [31,33-35].

In our hands, the C57B/6 MOG35-55 EAE model is a model of axonal injury and inflammation, with little demyelination or oligodendrocyte death [36]. Here we report that axonal injury was reduced in *dab2*-deficient mice subjected to EAE. Multiple mechanisms could account for this observation. Firstly, it is believed that reactive oxygen species produced by microglia induce mitochondrial dysfunction, resulting in energy failure and axonal injury in MS [37]. Therefore, it is possible that *dab2*-deficient mice that produced less *iNOS* experienced less mitochondrial dysfunction and thereby had a relative preservation of axons relative to their wild-type littermates subjected to EAE. Secondly, *dab2*-deficient EAE mice exhibited smaller inflammatory cord infiltrates and thus contained fewer total microglia and macrophages within their spinal cords as compared with their wild-type littermates. Past *in vitro* studies have demonstrated that microglia/macrophage-mediated axonal transection occurs via a contact-dependent mechanism [27]. Therefore, it is possible that, in *dab2*-deficient mice, axonal transection was reduced due to fewer numbers of activated microglia making contact with axonal segments.

This paper presents evidence that Dab2-dependent microglial/macrophage responses worsen inflammatory tissue damage in EAE. As we have also shown high levels of Dab2 protein expression in cells with a macrophage morphology in early acute MS lesions, this finding could be of relevance to human MS. We have also demonstrated that MS lesional Dab2 expression markedly decreases within late active lesions and that it is almost absent in chronic active lesions. Additionally, we showed that high levels of Dab2 expression were associated with marked axonal injury in MS, again consistent with our acute EAE data that demonstrated that *dab2*-deficient mice had significantly less axonal injury than their wild-type littermates when subjected to EAE. Haider and colleagues recently established that oxidative damage is most profound in active MS lesions in which there is a high density of macrophages/microglia [38]. Given that we found Dab2 expression to be greatest in early acute lesions, and given that we have found an association between *dab2* expression and *iNOS* expression, it is possible that *dab2* up-regulation could lead to increases in the expression of reactive oxygen species, thus contributing to oxidative damage caused to axons, myelin and oligodendrocytes in these earliest lesions.

During the course of this study, we aimed to identify signals responsible for the up-regulation of *dab2* expression. However, candidates tested did not result in increases in *dab2* expression. Therefore, we can conclude that *dab2* expression is not directly mediated via Toll-like receptor signalling, nor through IFNγ signalling pathways. We did find, however, that both TGFβ_1_ and all-*trans* retinoic acid were responsible for the down-regulation of *dab2* in macrophages/microglia, although this was not a *dab2*-specific response, but rather part of a broader anti-inflammatory response, as previously described [39-42].

In addition to regulating pro-inflammatory biochemical pathways, a further possibility is that Dab2 may regulate cell migration in the context of EAE, as reported in other experimental paradigms [8,14]. Although we did not find any evidence of a selective macrophage/microglial recruitment deficit to lesions in Dab2-deficient mice, we found that Dab2-deficient EAE tissue was less cell-dense than wild-type EAE tissue. The lower overall density of macrophages/microglia in Dab2-deficient tissue could partially explain the effect of Dab2-deficiency on EAE severity. Future studies will benefit from a myeloid-lineage specific Dab2-knockout mouse model to further interrogate the role of Dab2 in these cells in EAE.

It has been reported that *dab2* is expressed in peripheral T-lymphocytes, however its expression is restricted to a FOXP3-positive T-regulatory cell population [22]. However, to exclude the possibility that EAE disease severity was ameliorated in Dab2 deficient mice due to aberrant T-cell priming, activation or regulation, we performed passive transfer EAE experiments, transplanting T-cells from Dab2-deficient mice into wild-type mice. We were able to induce disease in all eight wild-type recipient mice demonstrating that the EAE phenotype observed in Dab2-deficient mice was not mediated by altered signalling in T-lymphocytes. Additionally, we did not find any co-localisation between Dab2 and CD3 antigens within T-lymphocytes that had migrated into CNS lesions in wild-type mice subjected to EAE.

In this study, we did not probe the potential role of Dab2 in astrocyte or oligodendrocyte biology. It is possible that Dab2 mediates responses to inflammatory stimuli in these cell types, however this requires further investigation.

## Conclusions

Collectively, the data presented in this paper demonstrate that Dab2 is up-regulated in macrophages/microglia in acute EAE lesions and in early acute MS lesions. In EAE, we have shown that this endogenous upregulation is harmful, exacerbating axonal injury and worsening disease severity and disease-associated mortality. The molecule could be a future therapeutic drug target in MS, but no Dab2-specific inhibitors are known at present.

## Methods

### Ethics approvals

All animal experimentation was conducted according to National Health and Medical Research Council guidelines (Australia) and with approval from the Howard Florey Institute animal ethics committee.

Human histological studies were conducted in the laboratory of co-author T. Kuhlmann. All human material was sourced from the Neuropathology archives at the University of Muenster. Biopsies were conducted to exclude malignancy, and none of the authors had any part in the clinical management of the patients. Evaluation of neuropathological material was conducted using codified samples and conducted under the governance and with ethics approvals of the University of Muenster.

### Animals and reagents

C57B/6 and SJL/J mice were obtained from the Animal Resource Centre (Canning Vale, Western Australia, Australia). The (129sv/C57B/6) Dab2 *loxP* flanked strain (Dab2^fl/fl^) was a kind gift from Dr. Johnathan Cooper of the Fred Hutchinson Cancer Research Centre (Seattle, WA) [8]. These mice were backcrossed for 12 generations to generate a Dab2 *loxP* flanked strain on a C57B/6 background. The Meox2-Cre line was obtained from The Jackson Laboratory (Bar Harbor, Maine) on a mixed background (129sv/C57B/6 backcrossed onto a C57B/6 background for six generations). These mice were further backcrossed on to a C57B/6 background for 8 generations. Dab2^fl/fl^ mice were crossed to Meox2-cre mice to generate animals in which Dab2 was deleted from the embryo, whilst preserving Dab2 expression within extra-embryonic tissue (Dab2 wild-type, Dab2 heterozygote, Dab2 knockout mice).

All chemicals were obtained from Sigma-Aldrich (St. Louis, MO) unless otherwise specified. All cell culture plasticware was purchased from Nalgene Nunc International (Rochester, NY). All cell culture media and reagents were purchased from Invitrogen (Carlsbad, CA). All secondary antibodies were purchased from Jackson ImmunoResearch Laboratories (West Grove, PA) unless otherwise noted.

### Experimental autoimmune encephalomyelitis (EAE) induction

EAE was induced in 8–12 week old male and female mice by subcutaneous injection into the flanks and base of tail of 125μg of MOG_35-55_ (MEVGWYRSPFSRVVHLYRNGK) or PLP_139-151_ (HSLGKWLGHPDKF) (Auspep, Australia) emulsified in Complete Freund’s Adjuvant containing 4 mg/ml *Mycobacterium tuberculosis* H37Ra (Difco, Detroit, MI). Mice also received an intraperitoneal (i.p) injection of 400 ng pertussis toxin (List Biological, Campbell, CA) on days 0 and 3 of induction. Assessments of EAE severity were performed daily according to a 9-point paraplegia scale with 0.5-point increments [43,44]. Mice that reached grade 3.5 were killed in accordance with ethics committee requirements. EAE cohort 1 comprised Dab2wt/wt (n=32) and Dab2wt/ko (n=37) mice. EAE cohort 2 comprised Dab2wt/wt (n=27) and Dab2ko/ko (n=16) mice. Fewer Dab2ko/ko mouse numbers were available due to the relative difficulty in deriving this genotype.

Unless specified otherwise, all mouse tissue was derived from mice 18 days post-EAE induction.

Non-parametric statistical analyses were used to determine statistical significance of biological phenotypes seen in EAE experiments. The Mann–Whitney Rank Sum Test was used to compare between 2 groups (Dab2wt/wt versus Dab2wt/ko or Dab2wt/wt versus Dab2ko/ko) of EAE challenged animals. Survival was analysed using Kaplan-Meier survival curves followed by Log Rank test statistical analyses.

### Microarray analysis of gene expression

Microarray studies were performed on whole spinal tissue from 8 week old SJL/J mice subjected to EAE, and from age and gender matched healthy control mice killed on the same day. All EAE mice were killed at a disease level of severe hindlimb paraparesis or complete hindlimb paraplegia (EAE grade 2.5-3.0). Mice were deeply anesthetized with sodium pentobarbital (100 mg/kg i.p) and intracardially perfused with 20 mls 0.1 M PBS. The spinal cords were removed and immediately snap frozen in liquid nitrogen, and then stored at −80°C until use. RNA was extracted using an RNeasy mini kit (QIAGEN Pty Ltd. Vic, AUS) as per manufacturer’s instructions. Samples were incubated with 600 μl buffer RLT and homogenized using a Dounce homogenizer. The total RNA from spinal cords of two animals were pooled prior to the labeling reaction. A total of three healthy control pair samples (6 mice, n = 3) were compared with a total of five EAE pair samples (10 mice, n = 5). The RNA was then hybridized onto the murine MG U74Av2 microarray, Affymetrix®, Santa Clara CA, following manufactures instructions.

Expression data was analysed using Partek® genomics suite, Missouri, USA. Affymetrix® annotation library MG_U74Av2.na31.annot.csv was used and Data was normalized using RMA (Bolstad et al. 2003) and quantile normalization and adjusted for GC content. Expression values were log transformed using base2 prior to differential expression analysis using a 2 way ANOVA on treatment and batch. Cel files and normalized expression data have been deposited at the Gene Expression Omnibus (GSE44989).

### Adoptive transfer EAE

Adoptive transfer EAE was performed as previously described [45]. Briefly, active MOG_35-55_ EAE was induced in 8 to 12 week old male and female Dab2 knockout (n=5) and Dab2 heterozygous (n=3) donor mice. On day 10 post-immunization, T-lymphocytes were isolated from the draining lymph nodes and spleens of these mice. Cells were cultured in complete DMEM containing 50 ug of MOG_35-55_ peptide and 20 ng of IL-2, for 48 h at 37C in a 5% CO_2_ incubator. After this time, the non-adherent T-lymphocytes were collected, washed twice with PBS containing 0.5% FBS, and injected into C57B/6 wild-type recipient mice at a concentration of 2x10^6^ cells in 0.1 ml PBS, i.p, per mouse (transfer ratio was approximately 1 donor to 1 recipient mouse). On the same day, recipient mice were injected with 300 ng pertussis toxin i.p. on the opposite side to the site of T-lymphocyte cell injection. Assessments of disease severity were performed daily for 22 days as described above.

### Histology and immunohistochemistry

#### Mouse

Mice were anesthetized and intracardially perfused with MT-PBS followed by 4% paraformaldehyde. The lumbar expansions of spinal cords were isolated and equilibrated in 20% sucrose prior to embedding in OCT. Spinal cords were then sectioned at 10 μm intervals and collected onto chrom-alum-coated slides.

To detect murine Dab2, sections were rehydrated in MT-PBS and post-fixed in ice-cold acetone/methanol 50%/50% v/v solution for two minutes. Sections were washed 3x in MT-PBS, and blocked in 10% (v/v) normal goat serum (NGS) in MT-PBS with 0.5% BSA and 0.3% (v/v) TritonX-100 for 1hr at RT. Sections were labelled with rabbit anti-Dab2 (1:100; Santa Cruz Biochemicals, Santa Cruz, CA) alone, or in combination with rabbit anti-NG2 (1:200; Chemicon, Billerica, MA), mouse anti-GFAP (1:500; Chemicon), PE-CD11b antibody (2 μg/ml; CALTAG, Carlsbad, CA), or IgG2b-PE isotype control (2 μg/ml, BD, Franklin Lakes, NJ), rabbit anti-CD3 (1:100, DAKO, Glostrup, Denmark), mouse anti-NeuN (1:500, Chemicon) for 3 hrs at RT. Appropriate fluorescently-labelled secondary antibodies against host species were used at a dilution of 1:500 for 30 minutes at RT. Images were acquired by confocal microscopy (Zeiss LSM5 PASCAL).

### Human biopsy and autopsy specimens

This study was approved by the ethics committee of the University of Münster.

### Classification of multiple sclerosis lesions

All lesions fulfilled the generally accepted criteria for the diagnosis of multiple sclerosis [46,47]. Demyelinating activity was classified as described in detail earlier [48].

Biopsy specimens were fixed in 4% paraformaldehyde and embedded in paraffin. Autopsy material was generally fixed in 10% formalin and tissue blocks containing lesions were embedded in paraffin. Biopsy and autopsy tissues were cut in 4 m thick sections and stained with haematoxylin and eosin, Luxol-fast blue and Bielschowsky’s silver impregnation for lesion classification.

After deparaffinization, antigen retrieval was performed using a slide steamer for 35 minutes with a solution of Tris-EDTA (pH 9.0). After cooling slides on ice and two water rinses, intrinsic peroxidase activity was blocked by incubation with 5% H2O2 in PBS for 20 min. Non-specific antibody binding was inhibited with 10% FCS in PBS for 20 min. For the Dab2 stain, this was followed by incubation with mouse anti-Dab2 IgG1, (BD, 610465) at 1/100, in the same block for 12 hours at 4 degrees. The stain was developed using avidin-biotin immunohistochemistry with a secondary anti-mouse biotinylated Ab and the Vector ABC kit according to the manufacturer’s instructions, followed by the DAB reaction, which was terminated after 8 minutes. Controls omitting the primary Ab showed no staining. The slides were briefly dipped in haematoxylin solution for counter-staining before mounting.

For the APP/CD68 double stain, the sections were first incubated with mouse anti-human APP Ab (Chemicon MAB348) at 1/2000 in block at 4 degrees for 12 hrs, followed by secondary anti-mouse Ab conjugated to alkaline phosphatase, which was visualised using NBT/BCIP. This was followed by incubation with mouse anti-human CD68 (DAKO, M0876) at 1/100 in block for 12 hrs at 4 degrees followed by secondary goat anti-mouse IgG3-HRP (ABD serotec, STAR136P) at 1/200 for one hour, followed by DAB development and brief haematoxylin couterstain followed by mounting.

### Morphometry for human sections

Cell numbers and APP positive elements are expressed as an average number per 10 000 mm^2^ using 40x magnification. Averages were obtained by counting at least 6, and if possible, 10 standardised fields of view per lesion of 10 000 mm^2^ each defined by an ocular morphometric grid as previously described [25]. Fields for quantitating acute lesions were selected from within the heavily macrophage-infiltrated areas of the biopsies and, for the chronic active lesions, the edge of the lesions containing macrophages/microglia was selected.

### Quantitative PCR

cDNA was generated from 1μg of RNA using Taqman Reverse Transcription reagents (Applied Biosystems, Foster City, CA) according to manufacturer’s instructions. Quantitative PCR (qPCR) reactions were performed using Cybergreen PCR mastermix (Applied Biosystems) on a 7500 Fast Real Time PCR System (Applied Biosystems) using the comparative Ct method (Livak & Schmittgen, 2001). qPCR primers were designed using Primer Express 3.0 (Applied Biosystems). Primer sequences were as follows: mouse 18S, forward 5′CGGCTACCACATCCAAGGAA3′, reverse 5′GCTGGAATTACCGCGGCT3′; mouse Dab2 (exon 3 to detect p96 and p67) forward 5′TGTTGGCCAGGTTCAAAGGT3′, reverse 5′GCACATCATCAATACCGATTAGCT3′; mouse IFNγ forward 5′TTGGCTTTGCAGCTCTTCCT3′, reverse 5′TGACTGTGCCGTGGCAGTA3′; mouse iNOS forward 5′GGATCTTCCCAGGCAACCA3′, reverse 5′AATCCACAACTCGCTCCAAGATT3′. qPCR Ct values were normalised to 18S [49]. A dissociation step was performed to ensure that the signal produced was specific to the generation of a PCR product, and not due to primer dimerization.

Statistical significance was tested using the Spearman’s correlation co-efficient to analyse the correlation between EAE grade and fold increase of *dab2*. One-Way ANOVA with a 95% confidence interval, followed by Tukey’s *post hoc* comparison was used to test the expression of Dab2 at varying disease grades. Multivariate linear regression analysis was used to analyse the correlation between Dab2 expression and the expression of pro-inflammatory mediators in the spinal cords of Dab2 wild-type and Dab2 heterozygous mice subjected to EAE whilst controlling for EAE score.

### Western blotting

Protein lysates (100μg) together with pre-stained protein standards (Bio-Rad, Hercules, CA) were run on 8% Novex Tris-Glycine gels (Invitrogen). Proteins were transferred onto PVDF membranes (Pall Corporation, Port Washington, NY), blocked with 5% skim milk and probed with mouse anti-Dab2 (1:500; BD) at 4°C overnight (O/N). Membranes were washed 4x, followed by incubation with a goat-anti mouse HRP-conjugated secondary antibody (Upstate, Billerica, MA) for 1 hour at RT. Membranes were washed 6x prior to exposure to an enhanced chemiluminescence detection system (Amersham, Fairfield, CT). Signal detection was performed on a FUJIFILM LAS3000 imaging system, using LAS-3000 imaging software.

### Histological analysis of EAE disease severity

Inflammation analyses were performed by examining at least four, 10 μm sections per animal taken 50 μm apart to cover a minimum cross-sectional area of 200 μm of the lumber expansion of the spinal cord. Spinal cord area (μm^2^), the number of discrete immune infiltrates per spinal cord section and the size of each individual infiltrate (μm^2^) were analysed by an experimenter blinded to the genotype of the mice. Lesions were defined as areas of dense accumulation of Hoechst-positive nuclei both within the white and grey matter. All results are presented as average ± SEM. Statistical significance was tested using One-Way ANOVA with a 95% confidence interval, followed by Tukey’s *post hoc* test.

Lesion composition analysis was performed on three lesions per transverse section per mouse within the lumbar expansion of the spinal cord. To detect cell-specific markers, 10μm sections were rehydrated in MT-PBS and then blocked in 10% (v/v) NGS in MT-PBS with 0.5% BSA and 0.3% (v/v) TritonX-100 for 1hr at RT. Serial sections were labelled with primary antibodies directed against the microglial marker ionized calcium-binding adaptor molecule-1 (Iba1, 1:1,000; Wako Pure Chemicals, Tokyo, Japan); the astrocytic marker GFAP (1:500; Chemicon); and the oligodendrocyte precursor cell marker NG2 (1:200; Chemicon) in blocking solution at 4°C O/N. Appropriate fluorescently-labelled secondary antibodies against host species were used at a dilution of 1:500 for 1 hour at RT. Sections were also labelled with Hoescht 33342 (1:5,000; Invitrogen) for nucleus detection. Images were obtained using an upright microscope (Zeiss Axioplan 2) at x40 magnification. Images were opened in Adobe Photoshop CS3 and cropped such that the region of interest for each lesion was consistent between cell-specific stains. Regions of interest contained only cells constituting the core of the lesion. Counts of immuno-positive cells with nuclei were determined for each image and expressed as cells/mm^2^. All counts and analyses were performed blinded to the genotype of the animal. All results are presented as average ± SEM. Statistical significance was tested using One-Way ANOVA with a 95% confidence interval, followed by Tukey’s *post hoc* test.

### pNF-H ELISA

Phosphorylated neurofilament heavy chain (pNF-H) enzyme-linked immuno-sorbent assay (ELISA) analyses were performed using a chicken anti-pNF-H antibody (EnCor Biotechnology, Gainesville, FL) as previously described in detail [16]. Statistical significance was tested using a Two-tailed Student’s t-test with a 95% confidence interval.

### Isolation and culture of mouse microglia

Primary mixed glial cell cultures were prepared from the brains of P1.5 C57B/6, CD11b-cre Dab2 knockout or Meox2-cre Dab2 knockout mouse pups by exploiting differential adhesion to plastic. In brief, mice were anesthetized with isofluorane, decapitated and brains removed to HBSS (Invitrogen) containing 1mM sodium pyruvate, 10 mM HEPES, 0.14% glucose, 0.3% BSA and 1.16 mM MgSO_4_. The meninges and choroid plexus were removed; the whole brain was minced and then digested in 0.015% trypsin (w/v) for 15 minutes. The trypsin reaction was stopped with the addition of 0.05% trypsin inhibitor (w/v). Cells were briefly centrifuged and resuspended in DMEM containing 1 mM sodium pyruvate, 0.05% insulin and 10% foetal calf serum (FCS). Cells were triturated to a single cell suspension, plated in PDL-coated 75 cm^2^ tissue culture flasks and cultured at 37°C/5% CO_2_. Culture media was changed on days 1 and 7 of culture. Microglia were isolated on day 13 by gentle percussion and collected together with the glial conditioned media (GCM). Cells were briefly centrifuged and plated on 6cm^2^ tissue culture dishes for 24 h in 50% GCM/50% Macrophage-SFM (Invitrogen), 0.5% FCS to allow cells to quiesce and ramify before experimentation. After 24 h, media was replaced with 100% Macrophage-SFM/0.5% FCS for 16 hours and cultured at 37°C/5% CO_2_. For assessment of microglial activation cells were either treated with D-PBS (control) or 1 μg/ml LPS for 24 hours prior to RNA isolation.

### Isolation and culture of mouse bone marrow macrophages

Mice were given a lethal dose of sodium pentobarbital (100 mg/kg i.p), and immediately following death, the femur and tibia were excised with fine scissors. Using a 23G needle and syringe filled with DMEM (Gibco), the bone marrow was flushed into a tube containing 40 ml of cold DMEM. Cells were pelleted by centrifugation at 350 x g for 5 min, and then resuspended at 1x10^6^ cells/ml in macrophage medium (DMEM, 10% FCS, CSF-1, 100 U/ml penicillin, 100 ug/ml streptomycin, 2 mM GlutaMax-1) and seeded onto a non-coated T75 tissue culture flasks for 3 days in a 5% CO2 incubator at 37°C. After 3 days, non-adherent cells were collected and centrifuged at 350xg for 7 min. The cells were then resuspended in macrophage media and plated at a density of 1.5 million cells/per well onto non-coated 6-well plates (IWAKI). To promote macrophage differentiation, MCSF (2.5 ng/ml) was also added to the media, and the cells were incubated for 4 days. The day before experiments (day 8 following isolation), a full media change was performed (including the addition of MCSF), and cells were left to settle overnight.

### Statistical analyses

Individual statistical tests were performed as described in relevant sections with a minimum alpha level of 0.8. All reported p values are two-tailed and for each analysis p<0.05 was considered significant. Statistical analyses were performed using SigmaStat 2.03 (Systat Software Inc, San Jose, CA). Multivariable linear regression analyses adjusting for EAE disease severity were performed using Stata version 12.0 (StataCorp College Station, Texas).

## Competing interests

The authors declare that they have no competing interests.

## Authors’ contributions

VGJ conceived, designed, performed the experiments (unless otherwise stated), and wrote the manuscript. MMG aided in EAE induction and scoring, performed the passive transfer EAE experiments, aided in isolating bone marrow macrophages, and aided in revising the manuscript. DAK aided in EAE induction, scoring and histochemical staining. WD aided in genotyping, isolating bone marrow macrophages and performed the in bone marrow macrophage gene expression analyses. VMP analysed and curated the microarray analyses and aided in revising the manuscript. TLC performed original EAE experiments in SJL/J mice for microarray analysis. AJ aided in passive transfer EAE experiments and bone marrow macrophage isolations, and in revising the manuscript. GS provided reagents for pNFH ELISA experiments and aided in revising the manuscript. TK provided access to human biopsy and autopsy specimens, aided in human histochemical staining, characterised stained human tissue and aided in revising the manuscript. TJK conceived and designed the experiments and revised the manuscript. HB conceived and designed the experiments, performed the human histochemical staining, aided in EAE induction and scoring and revised the manuscript. All authors read and approved the final manuscript.

## Authors’ information

Denotes equal last authorship: Trevor J Kilpatrick and Helmut Butzkueven.

## Supplementary Material

Additional file 1: Figure S1 Characterization of Dab2 expression by oligodendroglia within the EAE spinal cord. Immunofluorescence staining for Dab2 in the EAE grade 3.0 spinal cord of a C57/B6 wild-type mouse. Representative confocal images show a: co-localisation of Dab2 within a subpopulation of PLPdsRed-expressing oligodendrocytes in an inflammatory lesion (arrows). b: co-localisation of Dab2 within a subpopulation of NG2-positive oligodendrocyte progenitor cells (arrow). Scale bars represent 50 μm. Click here for file

Additional file 2: Figure S2 *In vitro* expression of Dab2 in primary glia. a: Immunoprecipitation-Western blot analysis shows that oligodendrocyte progenitor cells,mature oligodendrocytes, astrocytes and microglia express both the p96 and p67 isoforms of Dab2. The p96 isoform is expressed more highly in astrocytes, whereas the p67 isoform is more highly expressed in microglia. b: *dab2* mRNA is expressed by cultured primary glial cells, with the highest levels in microglia, with diminishing expression by astrocytes, oligodendrocyte precursors and mature oligodendrocytes. ttest *p<0.05, **p<0.01. Click here for file

Additional file 3: Figure S3 Histological analysis of NAWM the lumbar expansion in Dab2wt/wt, Dab2wt/ko and mice subjected to EAE and in the dorsal column of healthy mice. A: Tissue was derived from mice at 18 days post-EAE induction. Cell densitometry analysis within normal-appearing white matter (NAWM) reveals that there are no significant differences between the proportions of Iba1+ microglia, GFAP+ astrocytes, and NG2+ oligodendrocyte progenitors in wild-type and Dab2 heterozygous mice within these regions. Total cell numbers within the NAWM of Dab2 heterozygous mice, however, are significantly fewer than in the NAWM of wild-type littermates (Average ± SEM; two-tailed student’s t-test, p<0.01). B: Analysis of cell densities within the dorsal column of Dab2 wild-type, heterozygote and knock-out mice reveals that there are no significant differences in the densities of GSTpipositive oligodendrocytes, Iba1-positive microglia, GFAP-positive astrocytes, NG-2 progenitors nor in total cell densities (n=3,4,3 animals/genotype respectively; Average ± SEM; One- Way ANOVA, p>0.05) 3. Click here for file

Additional file 4: Figure S4 Dab2-deficient T-cells can induce EAE in wild-type mice T-cells derived from EAE-induced Dab2-deficient mice were able to passively transfer disease in C57B/6 wildtype mice. Disease was induced in 5/5 wild-type recipients after transfer of T-cells from Dab2 knockout mice, and in 3/3 wild-type recipients after transfer of T-cells from Dab2 heterozygote mice. There were no significant differences between groups (Mann-Whitney rank sum test, p>0.05 at all time points). Click here for file

Additional file 5: Figure S5 In vitro modulation of Dab2 a: All-trans-Retinoic Acid has a potent anti-inflammatory effect on gene expression profiles within primary microglia. dab2 and TNFα gene expression are significantly down-regulated at 6 and 24 hours post-treatment with 1 μM Retinoic Acid (**p<0.01; ***p<0.001). b: TGFβ1 (10ng/ml) down-regulates dab2 and TNFα gene expression in primary microglia (*p<0.05; **p<0.01). a/b: n = 3 expts; Average ± SEM; One-Way ANOVA) c: Western blot probe for phosphorylated Dab2 p96 isoform and bactin loading control. Dab2 expressed by primary microglia is rapidly but transiently phosphorylated in response to TGFβ1. Phosphorylated Dab2 is present within 5 minutes of treatment, but no longer observed 30 minutes after the exogenous TGFβ1 pulse. Click here for file

Additional file 6: Figure S6 APP staining in MS Lesions a/b: APP staining for axonal injury (arrows) in the early active lesion (a: brown) and chronic acitve lesion (b: blue, CD68 brown) shows that axonal injury is much more pronounced in the early active lesion than in the chronic active lesion. Click here for file
