# Supplementary material for: Endogenously regulated Dab2 worsens inflammatory injury in experimental autoimmune encephalomyelitis
Source: Acta Neuropathol Commun. 2013 Jul 9;1:32. doi: 10.1186/2051-5960-1-32 (PMC3893401; doi:10.1186/2051-5960-1-32)
Supplement: Additional file 1: Figure S1 — Characterization of Dab2 expression by oligodendroglia within the EAE spinal cord. Immunofluorescence staining for Dab2 in the EAE grade 3.0 spinal cord of a C57/B6 wild-type mouse. Representative confocal images show a: co-localisation of Dab2 within a subpopulation of PLPdsRed-expressing oligodendrocytes in an inflammatory lesion (arrows). b: co-localisation of Dab2 within a subpopulation of NG2-positive oligodendrocyte progenitor cells (arrow). Scale bars represent 50 μm. [file 2051-5960-1-32-S1.pdf]

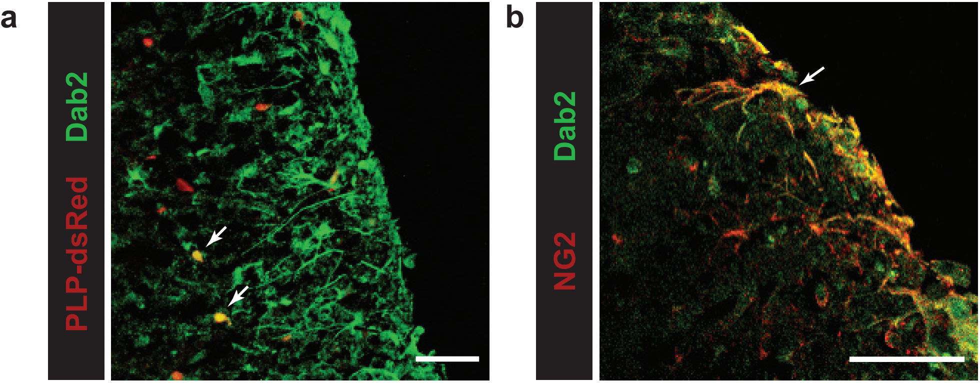

Figure S1 Characterization of Dab2 expression by oligodendroglia within the EAE spinal cord
Immunofluorescence staining for Dab2 in the EAE grade 3.0 spinal cord of a C57/B6 wild-type mouse. Representative confocal images show **a:** co-localisation of Dab2 within a subpopulation of PLPdsRed-expressing oligodendrocytes in an inflammatory lesion (arrows). **b:** co-localisation of Dab2 within a subpopulation of NG2-positive oligodendrocyte progenitor cells (arrow). Scale bars represent 50 μm.
