# Supplementary material for: Endogenously regulated Dab2 worsens inflammatory injury in experimental autoimmune encephalomyelitis
Source: Acta Neuropathol Commun. 2013 Jul 9;1:32. doi: 10.1186/2051-5960-1-32 (PMC3893401; doi:10.1186/2051-5960-1-32)
Supplement: Additional file 2: Figure S2 — In vitro expression of Dab2 in primary glia. a: Immunoprecipitation-Western blot analysis shows that oligodendrocyte progenitor cells,mature oligodendrocytes, astrocytes and microglia express both the p96 and p67 isoforms of Dab2. The p96 isoform is expressed more highly in astrocytes, whereas the p67 isoform is more highly expressed in microglia. b: dab2 mRNA is expressed by cultured primary glial cells, with the highest levels in microglia, with diminishing expression by astrocytes, oligodendrocyte precursors and mature oligodendrocytes. ttest *p<0.05, **p<0.01. [file 2051-5960-1-32-S2.pdf]

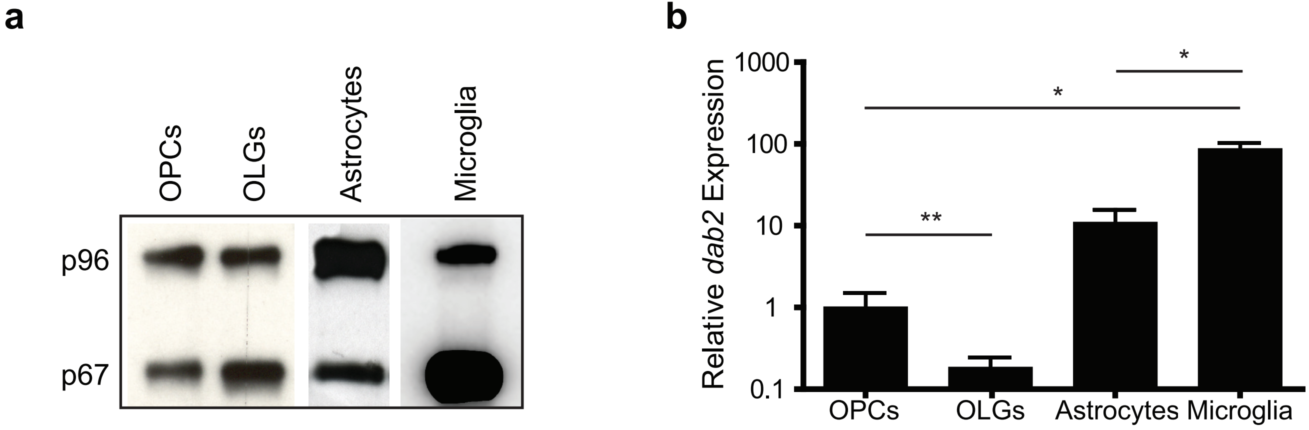

Figure S2 *In vitro* expression of Dab2 in primary glia

a: Immunoprecipitation-Western blot analysis shows that oligodendrocyte progenitor cells, mature oligodendrocytes, astrocytes and microglia express both the p96 and p67 isoforms of Dab2. The p96 isoform is expressed more highly in astrocytes, whereas the p67 isoform is more highly expressed in microglia. **b:** *dab2* mRNA is expressed by cultured primary glial cells, with the highest levels in microglia, with diminishing expression by astrocytes, oligodendrocyte precursors and mature oligodendrocytes. ttest $^{*}p<0.05$, $^{**}p<0.01$.
