# Supplementary material for: Endogenously regulated Dab2 worsens inflammatory injury in experimental autoimmune encephalomyelitis
Source: Acta Neuropathol Commun. 2013 Jul 9;1:32. doi: 10.1186/2051-5960-1-32 (PMC3893401; doi:10.1186/2051-5960-1-32)
Supplement: Additional file 3: Figure S3 — Histological analysis of NAWM the lumbar expansion in Dab2wt/wt, Dab2wt/ko and mice subjected to EAE and in the dorsal column of healthy mice. A: Tissue was derived from mice at 18 days post-EAE induction. Cell densitometry analysis within normal-appearing white matter (NAWM) reveals that there are no significant differences between the proportions of Iba1+ microglia, GFAP+ astrocytes, and NG2+ oligodendrocyte progenitors in wild-type and Dab2 heterozygous mice within these regions. Total cell numbers within the NAWM of Dab2 heterozygous mice, however, are significantly fewer than in the NAWM of wild-type littermates (Average ± SEM; two-tailed student’s t-test, p<0.01). B: Analysis of cell densities within the dorsal column of Dab2 wild-type, heterozygote and knock-out mice reveals that there are no significant differences in the densities of GSTpipositive oligodendrocytes, Iba1-positive microglia, GFAP-positive astrocytes, NG-2 progenitors nor in total cell densities (n=3,4,3 animals/genotype respectively; Average ± SEM; One- Way ANOVA, p>0.05) 3. [file 2051-5960-1-32-S3.pdf]

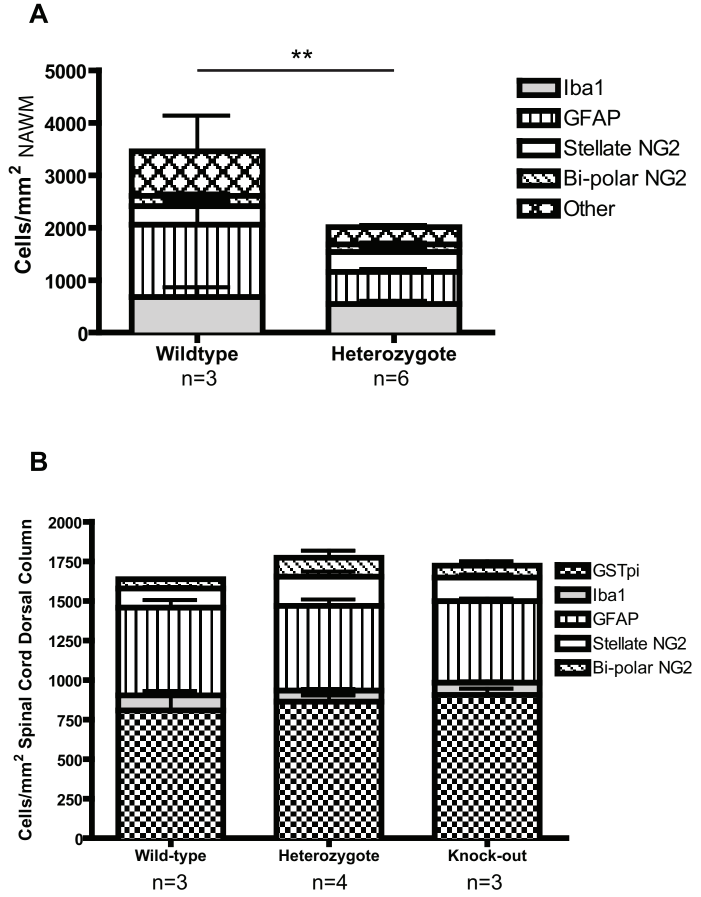

Figure S3 Histological analysis of NAWM the lumbar expansion in Dab2wt/wt, Dab2wt/ko and mice subjected to EAE and in the dorsal column of healthy mice.

A: Tissue was derived from mice at 18 days post-EAE induction. Cell densitometry analysis within normal-appearing white matter (NAWM) reveals that there are no significant differences between the proportions of Iba1+ microglia, GFAP+ astrocytes, and NG2+ oligodendrocyte progenitors in wild-type and Dab2 heterozygous mice within these regions. Total cell numbers within the NAWM of Dab2 heterozygous mice, however, are significantly fewer than in the NAWM of wild-type littermates (Average ± SEM; two-tailed student's t-test, $p<0.01$).

B: Analysis of cell densities within the dorsal column of Dab2 wild-type, heterozygote and knock-out mice reveals that there are no significant differences in the densities of GSTpi-positive oligodendrocytes, Iba1-positive microglia, GFAP-positive astrocytes, NG-2 progenitors nor in total cell densities (n=3,4,3 animals/genotype respectively; Average ± SEM; One-Way ANOVA, $p>0.05$).
