# Supplementary material for: Endogenously regulated Dab2 worsens inflammatory injury in experimental autoimmune encephalomyelitis
Source: Acta Neuropathol Commun. 2013 Jul 9;1:32. doi: 10.1186/2051-5960-1-32 (PMC3893401; doi:10.1186/2051-5960-1-32)
Supplement: Additional file 4: Figure S4 — Dab2-deficient T-cells can induce EAE in wild-type mice T-cells derived from EAE-induced Dab2-deficient mice were able to passively transfer disease in C57B/6 wildtype mice. Disease was induced in 5/5 wild-type recipients after transfer of T-cells from Dab2 knockout mice, and in 3/3 wild-type recipients after transfer of T-cells from Dab2 heterozygote mice. There were no significant differences between groups (Mann-Whitney rank sum test, p>0.05 at all time points). [file 2051-5960-1-32-S4.pdf]

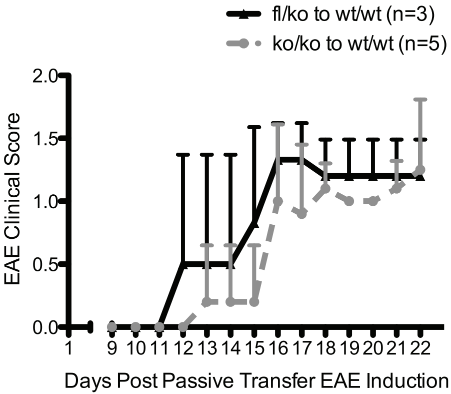

Figure S4 Dab2-deficient T-cells can induce EAE in wild-type mice

T-cells derived from EAE-induced Dab2-deficient mice were able to passively transfer disease in C57B/6 wildtype mice. Disease was induced in 5/5 wild-type recipients after transfer of T-cells from Dab2 knockout mice, and in 3/3 wild-type recipients after transfer of T-cells from Dab2 heterozygote mice. There were no significant differences between groups (Mann-Whitney rank sum test, $p>0.05$ at all time points).
