# Supplementary material for: Endogenously regulated Dab2 worsens inflammatory injury in experimental autoimmune encephalomyelitis
Source: Acta Neuropathol Commun. 2013 Jul 9;1:32. doi: 10.1186/2051-5960-1-32 (PMC3893401; doi:10.1186/2051-5960-1-32)
Supplement: Additional file 5: Figure S5 — In vitro modulation of Dab2 a: All-trans-Retinoic Acid has a potent anti-inflammatory effect on gene expression profiles within primary microglia. dab2 and TNFα gene expression are significantly down-regulated at 6 and 24 hours post-treatment with 1 μM Retinoic Acid (**p<0.01; ***p<0.001). b: TGFβ1 (10ng/ml) down-regulates dab2 and TNFα gene expression in primary microglia (*p<0.05; **p<0.01). a/b: n = 3 expts; Average ± SEM; One-Way ANOVA) c: Western blot probe for phosphorylated Dab2 p96 isoform and bactin loading control. Dab2 expressed by primary microglia is rapidly but transiently phosphorylated in response to TGFβ1. Phosphorylated Dab2 is present within 5 minutes of treatment, but no longer observed 30 minutes after the exogenous TGFβ1 pulse. [file 2051-5960-1-32-S5.pdf]

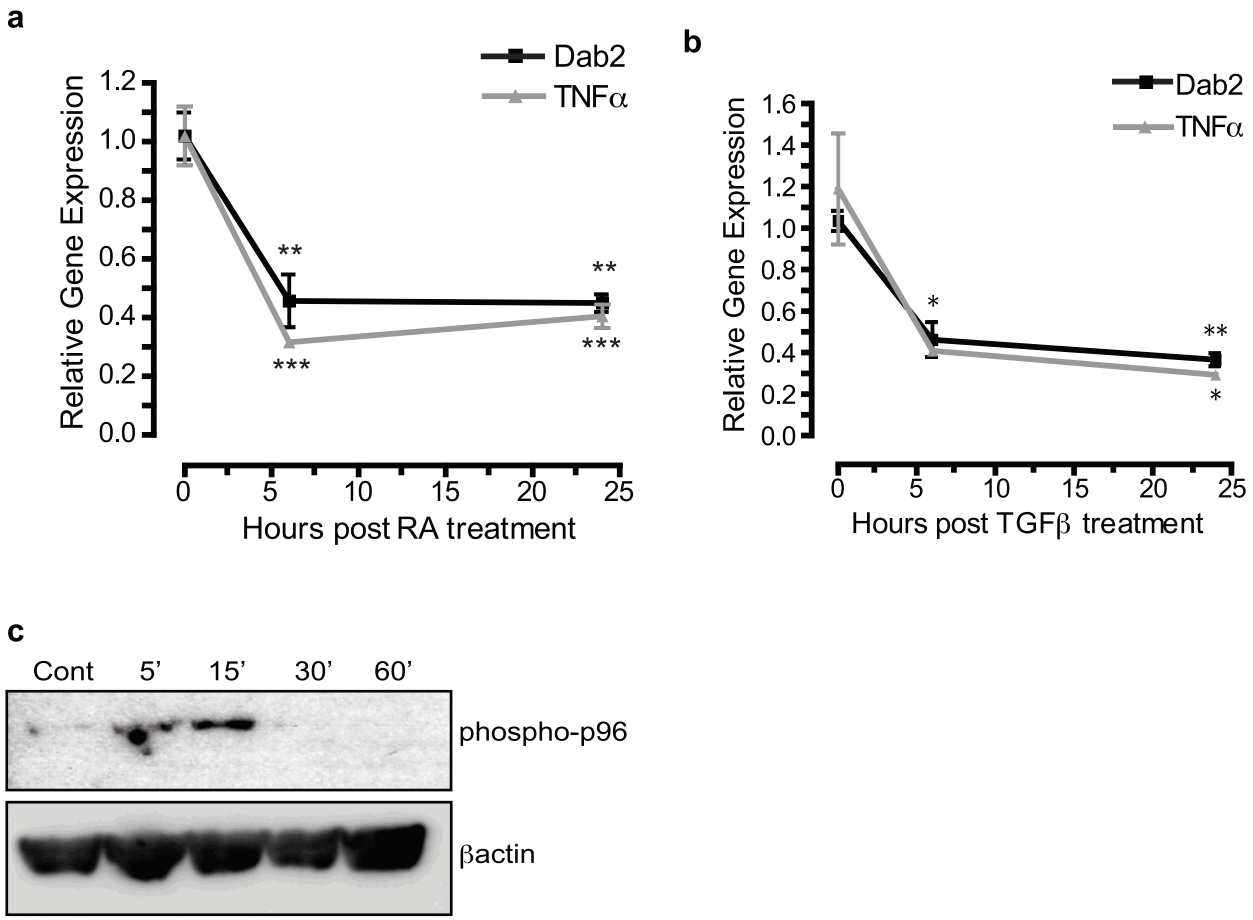

Figure S5 In vitro modulation of Dab2

a: All-trans-Retinoic Acid has a potent anti-inflammatory effect on gene expression profiles within primary microglia. dab2 and TNFα gene expression are significantly down-regulated at 6 and 24 hours post-treatment with 1µM Retinoic Acid (**$p<0.01$; ***$p<0.001$). b: TGFβ1 (10ng/ml) down-regulates dab2 and TNFα gene expression in primary microglia (*$p<0.05$; **$p<0.01$). a/b: n = 3 expts; Average ± SEM; One-Way ANOVA) c: Western blot probe for phosphorylated Dab2 p96 isoform and bactin loading control. Dab2 expressed by primary microglia is rapidly but transiently phosphorylated in response to TGFβ1. Phosphorylated Dab2 is present within 5 minutes of treatment, but no longer observed 30 minutes after the exogenous TGFβ1 pulse.
