# Supplementary material for: Endogenously regulated Dab2 worsens inflammatory injury in experimental autoimmune encephalomyelitis
Source: Acta Neuropathol Commun. 2013 Jul 9;1:32. doi: 10.1186/2051-5960-1-32 (PMC3893401; doi:10.1186/2051-5960-1-32)
Supplement: Additional file 6: Figure S6 — APP staining in MS Lesions a/b: APP staining for axonal injury (arrows) in the early active lesion (a: brown) and chronic acitve lesion (b: blue, CD68 brown) shows that axonal injury is much more pronounced in the early active lesion than in the chronic active lesion. [file 2051-5960-1-32-S6.pdf]

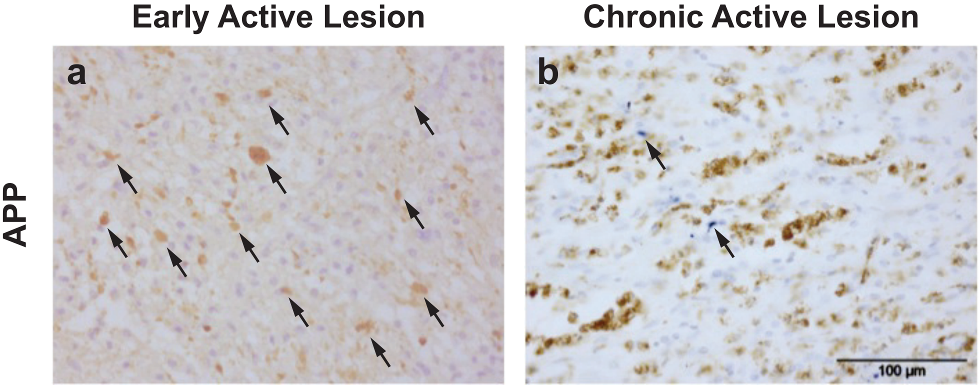

Figure S6 APP staining in MS Lesions
a/b: APP staining for axonal injury (arrows) in the early active lesion (**a:** brown) and chronic acitve lesion (**b:** blue, CD68 brown) shows that axonal injury is much more pronounced in the early active lesion than in the chronic active lesion.
